# Support interventions for families facing parental life-threatening illness – A scoping review

**DOI:** 10.1017/S1478951526101837

**Published:** 2026-04-14

**Authors:** Nina Malmström, Anneli Ozanne, Stefan Nilsson, Joakim Öhlén, Birgitta Jakobsson Larsson

**Affiliations:** 1 Institute of Health and Care Sciences, Sahlgrenska Academy, University of Gothenburg, Gothenburg, Sweden; 2 Region Västra Götaland, Sahlgrenska University Hospital, Department of Neurology, Gothenburg, Sweden; 3 Centre for Person-Centred Care, University of Gothenburg, Gothenburg, Sweden; 4 Region Västra Götaland, Sahlgrenska University Hospital, Queen Silvia Children’s Hospital, Gothenburg, Sweden; 5 Region Västra Götaland, Sahlgrenska University Hospital, Palliative Centre, Gothenburg, Sweden; 6 Department of Neurology, Uppsala University Hospital, Uppsala, Sweden; 7 Department of Public Health and Caring Sciences, Uppsala University, Uppsala, Sweden

**Keywords:** Family support, children, neurology, terminally ill, scoping review

## Abstract

**Objectives:**

Despite the urgent need for support interventions for families facing parental life-threatening illness, research is limited – particularly in progressive neurological diseases. This scoping review aimed to systematically map existing interventions to inform the development of tailored support in the neurological context.

**Methods:**

A scoping review was conducted, including articles published between 2013 and 2025, identified through searches in PubMed, CINAHL, PsycINFO, and Web of Science, along with manual screening of reference lists. Extracted data were systematically charted and descriptively summarized.

**Results:**

Of 5172 articles, 15 were included, describing 6 unique interventions aimed at supporting children (0–25 years) and/or parents in families where a parent had a life-threatening illness. While cancer was the predominant diagnosis among ill parents, progressive neurological diseases, such as amyotrophic lateral sclerosis (ALS) and Huntington’s disease, were represented to a limited extent. The interventions targeted children (*n* = 4), parents in their parenting role (*n* = 4), or the entire family (*n* = 7) and were primarily based on psychosocial, psychoeducational, or peer support. Overall, the interventions were positively received by both children and parents and perceived as helpful in navigating their challenging life situations in various ways.

**Significance of results:**

This review confirms a particular lack of knowledge and tailored support for families affected by progressive neurological diseases. While support interventions for other life-threatening illnesses are also limited, those that exist may offer valuable insights to inform the development of support within neurological care contexts. The findings underscore the need for early, proactive, and accessible approaches that address both individual and family needs across the disease trajectory, aligning with core principles of high-quality palliative care.

## Introduction

When a parent is diagnosed with a life-threatening illness, the whole family may be affected and in need of support. However, knowledge about available support for these families remains limited – and in the context of progressive neurological disease almost non-existent – underscoring the importance of comprehensive mapping of existing research to guide future development.

Children of parents with life-threatening illnesses are at increased risk of health problems such as anxiety, depression, stress (Wray et al. 2022; Bergersen et al. 2024), and school difficulties (Sieh et al. 2013), with potential long-term consequences including suicide risk (Ong et al. 2021). In families affected by fatal progressive neurological diseases, children may struggle with frightening thoughts of loss and change, take on adult responsibilities, and experience social isolation (Malmström et al. 2024; Cooper et al. 2025). Their needs may also go unnoticed, as indicated in a study where children of parents with ALS rated their own health as poorer than perceived by their parents (Nilsson et al. 2025). Both ill parents and co-parents in these families face a demanding everyday life marked by worry, grief, and uncertainty (Malmström et al. 2025). Parents with a fatal progressive neurological or neuro-oncological disease describe difficulties in balancing their illness and parenting, evoking feelings of guilt and inadequacy due to their reduced emotional or physical presence (Loughan et al. 2022; Malmström et al. 2025). Co-parents often carry a dual burden – supporting both the ill parent and the children while grappling with their own difficult emotions (Bergem and Aamotsmo 2024; Billaud Feragen et al. 2025; Malmström et al. 2025). The behavioral, cognitive, and physical changes associated with these diseases – which may occur rapidly – can create complex and multifaceted challenges for the entire family (Masrori and Van Damme 2020; Pender et al. 2020; Stoker et al. 2022; Schaff and Mellinghoff 2023), including concerns about heredity and disrupted parent–child relationships (Loughan et al. 2022; Cooper et al. 2025; Malmström et al. 2025).

Although the need for improved support in families facing a parent’s fatal progressive neurological illness has been acknowledged (Malmström et al. 2023, 2025; Billaud Feragen et al. 2025; Cooper et al. 2025), research on existing interventions in this context remains scarce. Given the limited literature, it is necessary to scope support interventions for families affected by all types of parental life-threatening illness. Here, life-threatening illness refers to conditions with high risk of near-term death, typically involving palliative care needs. This broader approach may allow for a comprehensive mapping of available interventions, providing insight into how support is structured and delivered, as well as into the findings reported across studies. Such mapping is essential for understanding how the needs of children, ill parents, and co-parents have been, and may be, addressed – while also identifying forms of support particularly relevant to families living with progressive neurological disease. With a special interest in informing the development of tailored support for this group, the aim of this scoping review was to systematically map support interventions for families in which a parent has a life-threatening illness.

## Methods

### Design

This scoping review followed the methodological framework developed by Arksey and O’Malley (2005), refined by Levac et al. (2010), and was reported in accordance with the PRISMA-ScR checklist (Tricco et al. 2018).

### Search strategy

The literature search began with a systematic database search – conducted in collaboration with an expert librarian – completed on 12 February 2025, followed by a manual screening of reference lists. The databases searched were PubMed, CINAHL, PsycINFO, and Web of Science. The search was structured into 4 search blocks: type of support intervention, children, family relations, and diagnoses. Filters for publication year (2013–2025) and peer reviewed articles, when applicable, were used (Supplementary File 1).

### Eligibility criteria

The review included original peer-reviewed articles on support interventions for families where a parent had a life-threatening illness. While progressive neurological diseases were initially of particular interest, all incurable diseases with fatal outcomes were eligible. No strict diagnostic list or time-based definition was applied (see Supplementary File 1 for full search terms). The term “children” refers to individuals in the role of a child to a parent with a life-threatening illness, including minors, adolescents, and young adults up to the age of 25 years. The decision to include interventions targeting young adults aged 19–25, despite no longer being legally children, was based on evidence and clinical experience showing that many in this age group continue to live at home when a parent becomes life-threateningly ill. In such situations, they often take on caring roles and remain actively involved in family life (Kavanaugh et al. 2020b, 2021). Furthermore, research suggesting that brain development is not fully completed until the mid-20s supports the relevance of including this group within the broader definition of “children” used in this study (Sawyer et al. 2018).

Inclusion criteria were: parents with a life-threatening illness (as defined by the search strategy; Supplementary File 1), co-parents, children aged ≤25 years; randomized controlled trials (RCTs), non-RCTs, interventions evaluated quantitatively or qualitatively; pilot and feasibility studies, brief reports, and study protocols. One study protocol was excluded due to overlap with included publications. The inclusion period covered January 2013 to February 2025 to capture the most recent and relevant research.

Exclusion criteria included non-English publications, reviews, case studies, and gray literature (e.g., reports, dissertations) to ensure scientific quality through peer review.

### Selection of sources of evidence

**Figure 1.**
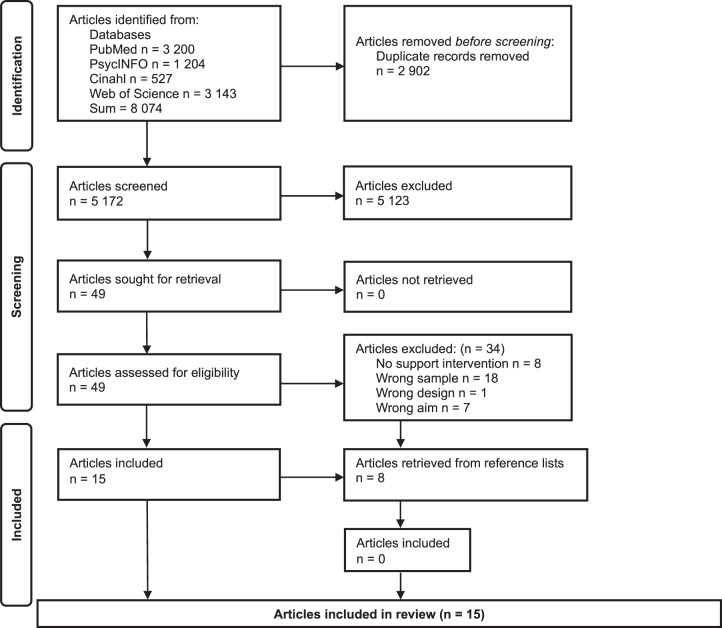
PRISMA 2020 flow diagram.

A total of 8074 citations were identified through the database search, of which 5172 remained after deduplication. A blinded screening of titles and abstracts, independently performed by the authors and 2 master’s students in the Rayyan software (Ouzzani et al. 2016) yielded 49 potentially relevant citations. Following full-text reading, 15 articles met the eligibility criteria and were selected for inclusion. Manual screening of reference lists from all 49 full-text articles yielded no additional inclusions (Figure 1).

### Data extraction and descriptive summary

Data relevant for the review aim were extracted in accordance with the stage of *charting the data* (Arksey and O’Malley 2005; Levac et al. 2010). Using a structured extraction sheet with predefined headings to capture details on the included articles and interventions, 2 authors independently extracted the data, which were reviewed by all authors and compiled into 2 tables (Tables 1 and 2). In line with the stage of *collating and summarizing the data*, the extracted data were then descriptively and narratively summarized. The articles were categorized based on the primary target group of the interventions, and each intervention was presented individually within its respective category to provide a structured overview of design, content, delivery, and reported findings.

**Table 1.** Overview of included articles, grouped by the intervention studied

**First author, year; country**	**Study design and analysis**	**Study sample**	**Study aim**	**Diagnosis**	**Main findings related to the review aim**	**Study quality**
**YCare Protocol**						
Kavanaugh et al. 2018; USA	Feasibility study (mixed methods); pre-post intervention self-report, module evaluations; no control group; descriptive statistics, narrative summary	4 children (8–18 y)	To assess the feasibility of a multidisciplinary young caregiver group training protocol for children and youths who provide care to a family member with ALS.	Amyotrophic lateral sclerosis (ALS)	**Quantitative findings:**	Medium
					The youths gave positive feedback, showing engagement with tasks and therapists. They reported learning new skills and correcting previously incorrect techniques.	
					**Qualitative findings:**	
					The model was feasible for both the youths and therapists. The final evaluation supported a peer engaged model; all the youths appreciated connecting with others in similar situations and many wanted to maintain contact. The therapists, despite no formal evaluation, deemed it a success based on active participation, willingness to engage with and teach back to the group, and observed positive behavioural changes.	
Kavanaugh et al. 2020b; USA	Pilot study (mixed methods); pre-post intervention self-report; no control group; descriptive and statistical analysis, narrative summary	19 children (9–19 y)	The study presents pilot data from pilot tests of the YCare protocol addressing the aims: young carer participants in the YCare protocol will show improved self-efficacy in care tasks after receiving the training; and youth participants can identify self-care goals and behaviors toward the development of self-management as caregivers.	Amyotrophic lateral sclerosis (ALS)	**Quantitative findings:**	Medium
					Increase in confidence in several care tasks (Caregiving Self-Efficacy Scale).	
					**Qualitative findings:**	
					Benefits of hands-on-training were highlighted, as well as the importance of meeting peers and professional adults.	
**Youth respite camp**						
Kavanaugh et al. 2017; USA and Canada	Evaluation study (mixed methods); pre-post intervention self-report; no control group; linear mixed model and descriptive statistics; thematic analysis	34 children (15–23 y)	To evaluate the effects of the camp, primarily by examining how the camp affected the youth’s feelings of isolation and their ability to receive support from others in similar situations.	Huntington’s disease (HD)	**Quantitative findings:**	Good
					Females showed increased self-esteem (Rosenberg’s Self-Esteem Scale) and resilience (Brief Resilience Scale) post-camp, while males’ resilience declined (Brief Resilience Scale). Social support improved across all participants, with the younger ones benefiting most. Informational support increased, especially among females. More than half of the participants reported greater knowledge about personal relationships and HD. Life satisfaction also significantly improved post-camp (Satisfaction with Life Scale).	
					**Qualitative findings:**	
					Participants reported feeling less alone, more positive, and better equipped to cope with HD. They also gained important information, helping some make decisions about testing.	
**My Kite Will Fly (MKWF)**						
Holland et al. 2018; Australia	Brief report (mixed methods); self-report, interviews; no control group; descriptive statistics, cross-case thematic analysis	36 children (2–13 y) 19 parents from 19 families	To develop an effective “resource toolbox” for multidisciplinary practitioners, both to deepen a child’s understanding of the complex physical and emotional processes involved in parental cancer treatments, and to equip parents with effective communication strategies.	Life threatening gynecological cancer	**Quantitative findings:**	Low
					At diagnosis, 86.1% rated high positive evaluation of MKWF. During treatment, 66.6% reported improved understanding of the treatment and better communication with the children. Among families participating in the tasks throughout palliative care, 97.3% found them crucial for stabilizing family routines. MKWF was considered adaptable to the patient’s preferences and family needs. While workbook evaluations were challenging to collect from the children due to varying coping abilities with parental illness, most children expressed great appreciation for their workbooks, regardless of whether they were in hardcopy or e-book format. **Qualitative findings:** MKWF was perceived to help both children and surviving parents to prepare for parental bereavement.	
**Family Audiobook**						
Cuhls et al. 2021; Germany, Austria and Benelux	Evaluation study (qualitative); post-intervention interviews; no control group; content analysis	44 ill parents with children <18 y	To evaluate an audiobook intervention for terminally ill parents with young children by qualified radio journalists.	Ill parents with short remaining life expectancy (predominantly cancer, but 1 with chronic obstructive pulmonary disease (COPD), 2 with amyotrophic lateral sclerosis (ALS), and 1 with Huntington’s disease (HD)	**Qualitative findings:** Recording the audiobook became a way for parents to create a lasting bond with their children, cope with their illness, and leave a personal legacy. They saw it as a source of comfort and strength for the children, even though the process was emotionally challenging. There were concerns about the children’s reactions to the audiobook after their death, but the hope was that it would provide support for them over time. The intervention also had a positive impact on family communication. All participants recommended the intervention and found it valuable.	Good
Ateş et al. 2024; Germany	Evaluation study (quantitative); post-intervention self-report; no control group; descriptive statistics; bivariate cross-tabulations	186 respondents: 95 terminally ill parents with children <18 y 91 individuals close to them (includes a diverse range of respondents, including partners, ex-partners, parents of patients, siblings, cousins, friends, bereaved children, and other relatives not further specified)	To examine how terminally ill parents and those close to them perceive the audiobook and how satisfied they are with it.	Terminal illness (predominantly cancer)	**Quantitative findings:** Almost all terminally ill parents felt eased to have recorded a family audiobook. Most ill parents (68%) listened to the audiobook immediately upon receiving it, and with predominantly ambivalent feelings (57%). Among children who had received the audiobook, 85% ill parents and close individuals stated that they had listened to it. Respondents valued the family audiobook as a cherished digital legacy and meaningful remembrance for the whole family, highlighting the personal stories and the lasting impact of hearing the voice of a deceased or dying parent.	Good
**Enhancing Connections-Palliative Care (EC-PC)**						
Lewis et al. 2020; USA	Pilot feasibility study (quantitative); pre-post intervention self-report; with comparison control group; descriptive and statistical analysis	26 ill parents (with 26 children, 5–8 y)	(1) Assess the feasibility of The Enhancing Connections-Palliative Care (EC-PC) parenting program (recruitment, enrollment, and retention); (2) test the short-term impact of the program on changes in parent and child outcomes; and (3) explore the relationship between parents’ physical and psychological symptoms with program outcomes.	Advanced or metastatic cancer and receiving noncurative treatment	**Quantitative findings:**	Good
					The study demonstrated that the program was feasible and well-received. Parents showed significant within-group improvements in self-efficacy related to supporting their child in coping with cancer, eliciting their cancer-related concerns, and providing emotional support. Between-group analyses showed improvements comparable to historical controls in parental anxiety (Spielberger State-Trait Anxiety Inventory), parenting self-efficacy (Help Child Scale), parenting skills (Parenting Skills Checklist), and children’s behavioral-emotional adjustment (internalizing, externalizing, and anxious/depressed subscales of the Child Behavior Checklist). No significant change in parenting quality (Disclosure of Negative Feelings Subscale); parental symptom distress (Condensed Memorial Symptom Assessment Scale) was unrelated to outcomes or dropout.	
Zahlis et al. 2020; USA	Pilot study (qualitative); post-intervention interviews; no control group; content analysis and constant comparative analysis	26 ill parents	To describe parents’ attributed gains in participating in a fully manualized psycho-educational program, the goals of which were to add to the parent’s interactional skills, competencies, and confidence in ongoing communication with their child about the parent’s incurable cancer.	Incurable cancer	**Qualitative findings:**	Good
					Gains from participating in the program: being ready for a conversation about my cancer; bringing things out in the open; listening better to my child; getting my child to open up; not getting in my child’s way; changing my parenting.	
**Family Talk Intervention (FTI)**						
Eklund et al. 2020; Sweden	Pilot study (mixed methods); post-intervention self-report, interviews; no control group; descriptive statistics and content analysis	25 children (8–17 y) from 20 families	To explore the potential effects of FTI from the perspectives of minor children whose parent is cared for in specialized palliative home care.	Life threatening illness (predominantly cancer)	**Quantitative findings:**	Good
					Among the younger children (8–12 y), 8/12 found it easier to talk to their mother, and 6/12 found it easier to talk to their father, while the rest reported no change in communication. Regarding sibling communication, half reported no change, 4/12 found it easier, and 1 child had no siblings. Among the older children (13–19 y), 7/11 reported unchanged communication with both their mother and father, and 8/11 with their siblings. The remainder experienced improvements. No children, regardless of age, reported worsened family communication after the intervention. After FTI, 8/12 younger children and 5/11 teenagers reported increased knowledge about their parent’s illness, while the rest did not experience any change. **Qualitative findings:** FTI increased the children’s knowledge about the parent’s illness. FTI was perceived to have improved family communication by facilitating more open conversation, understanding each other’s feelings and reactions, and gaining deeper insight into the family members’ experiences. The children also felt more prepared regarding the parent’s illness and the future. FTI gave them practical ideas about how to maintain communication within the family into everyday life.	
Alvariza et al. 2021; Sweden	Feasibility study (mixed methods); post-intervention self-report, interviews; no control group; descriptive statistics and content analysis	20 ill parents, 19 co-parents from 20 families with 52 children (2–25 y)	To evaluate the feasibility of FTI in terms of acceptability from the perspective of parents in families where a parent suffers from a life-threatening illness and receives specialized palliative home care.	Life threatening illness:	**Quantitative findings:**	Good
					83% of the parents reported that FTI fulfilled their expectations. 77% of ill parents and 94% of co-parents would recommend FTI to other families facing similar situations. 90% felt understood by the interventionists, and 86% were comfortable sharing their thoughts, although 19% of co-parents struggled with this. 55% found the timing appropriate, while 31% wished it had been offered earlier. 38% wanted additional meetings, 31% felt the number was sufficient, and 10% considered them too many. **Qualitative findings:** FTI’s structured yet flexible approach was appreciated but wishes for an earlier start in the illness trajectory and better preparation before the meetings were expressed. The ability to adapt meetings to family needs was valued, although long gaps between sessions sometimes reduced relevance. There were also some requests for separate sessions to discuss sensitive topics without the ill parent present. Interventionists played a crucial role, offering trust, support, and facilitating emotionally difficult conversations. FTI helped families express thoughts and emotions more openly, with separate meetings for parents and children supporting communication.	
				17 with cancer, 1 with advanced pulmonary disease, 1 with gastric bleeding due to Dieulafoy’s lesion, and 1 with chronic liver disease		
Eklund et al. 2022a; Sweden	Evaluation study (qualitative); field notes; no control group; interpretive description	20 families with 50 children (0–25 y)	To explore the child’s active participation in a family support program, the Family Talk Intervention, in accordance with Article 12 of the United Nations Convention on the Rights of the Child, when having a parent cared for in palliative care.	Life threatening illness with palliative diagnosis (17 with cancer, 3 parents with illnesses related to the lungs, liver, or intestines)	**Qualitative findings:**	Good
					All children were listened to and supported in freely raising topics they wanted to talk about during the intervention. However, only a quarter reached the minimum point required in Article 12, where their views were taken into account.	
		Meeting 3:				
		35 children				
		Meeting 5:				
		28 children				
		24 parents				
		Meeting 6:				
		8 children				
		22 parents				
Eklund et al. 2022b; Sweden	Feasibility study (mixed methods); post-intervention self-report, interviews; no control group; descriptive statistics and content analysis	25 children (8–17 y)	To explore the feasibility of the Family Talk Intervention (FTI) and its acceptability to dependent children when a parent is cared for in palliative home care.	Life threatening disease (predominantly cancer (13/16))	**Quantitative findings:**	Good
					20/23 felt understood by the interventionists and 17/23 found it easy to talk to them without their parents. 17/23 thought it felt good to talk to the interventionists without their parents, while 6/23 responded “I don’t know.” 17/23 found the number of meetings sufficient, although 5/23 wanted more. Among the teenagers, 10/11 felt that FTI was well-timed, entirely/partially met their expectations and allowed them to share their thoughts and feelings with interventionists. 10/11 would recommend FTI to others in similar situations.	
					**Qualitative findings:**	
					Overall, FTI was appreciated, with most children feeling seen, heard, and valued. They recommended it to others and appreciated the structure, though some wished for more meetings over a longer period. The intervention facilitated openness, but a few children found certain discussions overwhelming. The interventionists were trusted and perceived as caring, creating a safe space for expression. Many children experienced emotional relief from talking and found positive feedback from the interventionists to boost their self-esteem.	
Eklund et al. 2022c: Sweden	Pilot study (mixed methods); post-intervention self-report, field notes; no control group; descriptive statistics and thematic analysis	14 children (8–24 y) 7 ill parents 8 co-parents from 7 families	To explore how the family perceived information and communication about the imminent death during the illness trajectory and after the loss.	Life-threatening illness (exclusively cancer)	**Quantitative findings:**	Good
					The main findings described how families generally talked about death with each other, rather than about the intervention itself.	
					**Qualitative findings:**	
					Both parents and children perceived FTI as helpful for preparing and talking about the future, mainly by encouraging open communication.	
Weber Falk et al. 2022; Sweden;	Pilot study (mixed methods); pre-post intervention self-report, interviews; no control group; descriptive and statistical analysis, content analysis	20 ill parents, 19 co-parents from 20 families with 50 children (0–25 y)	To explore potential effects of FTI in specialized palliative home care from the ill parent’s and co-parent’s perspectives.	Life threatening illness (see Alvariza et al. 2021)	**Quantitative findings:**	Good
					A significant change was observed in the ill parents’ willingness to talk more about their illness at the first follow-up, as well as in their increased satisfaction with family conversations about the illness at the second follow-up. No other significant effects were found. **Qualitative findings:** Improved family communication and relationship; learned communication strategies that facilitated open sharing of thoughts and feelings; increased openness that led to better understanding of each other’s perspectives. Both ill parents and co-parents also experienced less worry, with ill parents specifically reporting a greater sense of security about the future.	
Bergersen et al. 2024; Sweden	Evaluation study (qualitative); long-term follow-up; post-intervention interviews; no control group; phenomenographic analysis	6 ill parents 3 co-parents from 9 families with children (4–22 y)	To describe the parents’ perceptions of the timing and length of FTI in relation to the illness trajectory, to explore what activities learnt by the FTI were still practiced in the long-term and what content of FTI was perceived as most valuable to coping in the long-term.	Life-threatening illness (exclusively cancer)	**Qualitative findings:** FTI was perceived to alleviate feelings of loneliness, and some families continued to use its communication tools even years after the intervention. FTI contributed to a more open expression of thoughts and feelings among children. Peer support received in conjunction with FTI provided important social and emotional benefits, both during and after the intervention. Interventionists were perceived as professionals who facilitated open and honest communication. FTI’s timing should be adapted to each family’s needs and situation, as some benefited from early participation, while others felt it came too soon or too late. There was also a need for extended support, as parents requested ongoing guidance after FTI ended (e.g. during and after deaths, deteriorating health, or occasional challenges for children in crisis).	Good

**Table 2.** Overview of studied interventions, grouped by the main target group (children, parents or the entire family)

**Intervention**	**Structure**	**Methods of delivered support**	**Intervention providers**
**First authors, year**			
**Child-based support**			
**YCare Protocol**	A multidisciplinary, 1-day caregiving training protocol for young caregivers in families with ALS, divided into 4 blocks (approx. 45 min/block).	Group training	A multidisciplinary team of therapists: physiotherapists, occupational therapists, speech therapists and social workers, and suppliers of assistive devices
Kavanaugh et al. 2018;			
Kavanaugh et al. 2020b	Block 1: basic care: transfer/gait training, toileting, dressing, rails, raised seats, shower		
	Block 2: speech communication: communication strategies, preparing food textures, cueing patient for swallowing, feeding		
	Block 3: assistive devices: BiPap, electric wheelchair, speech generating device (SGD)		
	Block 4: wrap-up		
**Youth respite camp** Kavanaugh et al. 2017	A 4-day camp for children and youths living in families with HD. Content centered on topics about grief and loss, being the best version of myself, testing, communication, relationships with family and/or friends, support, caregiving, and science and research. Primary focus of camp: create a peer supportive environment, with the goal of improving self-esteem, resilience, life satisfaction and living with HD.	Professionally led respite camp	Experienced HD health practitioners: 3 social workers, a nurse, and a psychiatrist
**My Kite Will Fly (MKWF)**	A systematic art therapy “template” for children of parents with life-threatening gynecological cancer. Creation of memorable workbooks with creative, reflective, and informative elements to support children’s emotional expression and processing, but also to provide parents with effective communication strategies, throughout the course of the illness.	Art therapy workbook activities	Multidisciplinary practitioners
Holland et al. 2018			
**Parent-based support**			
**Family Audiobook**	Professional recording of audiobooks by ill parents for their child(ren) as a lasting legacy, featuring narratives, music, readings, and other personally chosen content. Recording duration: a few hours up to 3 days.	Audiobook creation	Trained radio journalist
Cuhls et al. 2021;			
Ateş et al. 2024			
**Enhancing Connections-Palliative Care (EC-PC)**	A parenting program consisting of 5, manual-based, sessions (30–60 min) with 2-week intervals, supplemented with at-home assignments.	Telephone-based sessions	A trained nurse or a patient educator
Lewis et al. 2020; Zahlis et al. 2020	Session 1: anchoring yourself so you can help your child		
	Session 2: adding to your listening skills		
	Session 3: building on your listening skills and checking in with your child about the cancer		
	Session 4: becoming a detective in regard to your child’s coping and learning what helps your child		
	Session 5: anchoring your listening and detective skills and celebrating your success		
**Family-based support**			
**Family Talk Intervention (FTI)**	Six, manual-based meetings (1–2 h) at 1–2-week intervals to support open communication in families with a parent affected by severe illness.	In-person sessions	Two FTI-educated interventionists: a social worker and a deacon
Eklund et al. 2020; Alvariza et al. 2021; Eklund et al. 2022a; Eklund et al. 2022b; Eklund et al. 2022c; Weber Falk et al. 2022; Bergersen et al. 2024			
	Meetings 1, 2, 4: parents and interventionists		
	Meeting 3: child(ren) and interventionists		
	Meetings 5, 6: the whole family		

To map methodological strengths and limitations in the existing literature, all included articles were assessed using the Mixed Methods Appraisal Tool (MMAT), version 2018 (Hong et al. 2018). The assessment was conducted by the first author, following discussions with the co-authors to ensure transparency and consistency in interpretation. The overall quality ranking is presented in Table 1 (full appraisal in Supplementary File 2).

## Results

### Study characteristics

This review includes 15 articles from Sweden (*n* = 7), the USA (*n* = 4), Germany (*n* = 1), and Australia (*n* = 1). Two articles were multinational: 1 conducted in the USA and Canada, and 1 across Germany, Austria, and the Benelux region. Twelve of the articles were published within the last 5 years. Nine were mixed-method studies, 4 qualitative, and 2 quantitative. Nine of the articles were classified as feasibility or pilot studies, 5 as evaluation studies, and 1 as a brief report. Most studies were small in scale, and only 3 involved more than 40 participants. According to the MMAT appraisal (Hong et al. 2018), the methodological quality was generally good, although 2 studies were of medium quality and 1 of low quality (Table 1; Supplementary File 2).

Incurable cancer was the most commonly reported diagnosis among ill parents in the included studies. Progressive neurological diagnoses, such as ALS and Huntington’s disease, were also represented, though to a limited extent. Other diagnoses included were advanced pulmonary disease, chronic liver disease, and gastric bleedings due to Dieulafoy’s lesion. The interventions described in the articles primarily focused on psychosocial (*n* = 10), psychoeducational (*n* = 4), or peer support (*n* = 1), although several combined these approaches or included additional elements such as respite, creative activities, existential content, and practical support (Table 3).

**Table 3.** Primary and additional support components in the interventions, as identified in this review

**Intervention**	**Psychosocial support**	**Psychoeducational support**	**Peer support**	**Other elements**
**YCare Protocol**	✓	✓*	✓	None mentioned
**Youth respite camp**	✓	✓	✓*	Respite
**My Kite Will Fly (MKWF)**	✓*			Creative activities
**Family Audiobook**	✓*			Existential dimensions (e.g. life reflection, biography, legacy)
**Enhancing Connections-Palliative Care (EC-PC)**	✓	✓*		None mentioned
**Family Talk Intervention (FTI)**	✓*	✓	✓	Practical assistance

* Primary support component as described in the articles.

A descriptive summary was conducted to map the identified support intervention. Based on the primary target group, the articles were categorized into child-based (*n* = 4), parent-based (*n* = 4), and family-based (*n* = 7) interventions. The parent-based category comprised interventions directed toward either the ill parent, the co-parent, or both. With 4 interventions examined across multiple articles (*n* = 11), a total of 6 unique interventions are presented in this review. Quantitative findings in the included studies reflected preliminary effects at the group level, while qualitative findings captured participants’ subjective experiences and perceived impacts. An overview of the articles and categorization of interventions are presented in Tables 1 and 2, where the age groups of the children are also specified.

### Categorization of interventions

#### Child-based support interventions

Four articles described 3 interventions directed at children: a caregiving training protocol (Kavanaugh et al. 2018, 2020a), a respite camp (Kavanaugh et al. 2017), and the art therapy program My Kite Will Fly (MKWF) (Holland et al. 2018).

##### YCare Protocol

Two articles focused on an intervention aimed at supporting children providing care for a family member with ALS (Kavanaugh et al. 2018, 2020a). It was primarily centered on psychoeducation, designed to empower the children’s supportive roles through structured guidance, increased preparedness, and enhanced understanding. The intervention comprised a 1-day training course divided into 4 modules and was led by a multidisciplinary team. The focus areas of the modules were basic care, speech and feeding, assistive devices, and young caregiver support. By providing participants with information about the disease, care techniques, and practical skills training, the intervention intended to strengthen their ability to cope with their life situation. It sought to increase their knowledge, confidence, and coping skills while supporting them in dealing with the emotional and practical challenges of being a young caregiver. In addition to the educational sessions, the intervention included psychosocial and peer support through age-based small groups.

The intervention was associated with increased caregiving confidence, acquisition of new skills, and correction of previously incorrect techniques (Kavanaugh et al. 2018, 2020a). Children valued sharing experiences with peers and supporting each other through various tasks. The hands-on educational sessions were perceived to enhance their understanding of the disease and everyday coping, with practical demonstrations seen as more effective than verbal or written information alone (Kavanaugh et al. 2018). The intervention was also considered feasible by both children and providers.

##### Youth respite camp

One article focused on a 4-day respite camp aimed to foster a sense of community among children from families living with Huntington’s disease (Kavanaugh et al. 2017). Led by experienced health practitioners, the camp was designed to promote self-esteem, resilience, and life satisfaction through a range of activities and sessions. Children were divided into groups, with siblings placed separately but grouping those from the same geographic regions to encourage ongoing peer support. Sessions addressed topics such as grief and loss, self-image, genetic testing, communication, relationships, caregiving, and research. In addition to peer support, the intervention included psychosocial and psychoeducational components, while also providing respite from everyday challenges.

The camp was associated with improvements in social and informational support, life satisfaction, and disease-related knowledge, particularly among girls and younger children (Kavanaugh et al. 2017). Peer interactions were perceived to promote a sense of belonging and reduce loneliness, while participants described feeling more hopeful, less fearful about the future, and better equipped to cope with everyday challenges. Some also felt better informed when making decisions about genetic testing.

##### My Kite Will Fly (MKWF)

MKWF was an art therapy program developed to support children in emotional expression and processing when a parent is diagnosed with life-threatening gynecological cancer (Holland et al. 2018). The children participated in structured art therapy workbook activities during 3 phases of the illness: diagnosis, treatment, and palliative care. These included drawing, memory gathering, and exercises in communication and reflection. Although primarily aimed at children, MKWF also sought to support parents indirectly by providing them with tools to facilitate open family dialogue and thereby enhance their children’s understanding of the illness. In addition to its psychosocial focus, MKWF incorporated psychoeducational components, as the workbooks contained informative elements, together with creative activities.

MKWF was positively evaluated by most families at diagnosis and was associated with improved communication during treatment (Holland et al. 2018). Workbook tasks completed during the palliative phase were rated as critical for stabilizing family routines. Children appreciated the creative workbooks, while parents indicated feeling better equipped to share information with their children. Both children and surviving parents described the intervention as helpful in preparing for parental bereavement.

#### Parent-based support intervention

Four articles presented 2 parent-based interventions: an audiobook intervention (Cuhls et al. 2021; Ateş et al. 2024) and the telephone-delivered program Enhancing Connections-Palliative Care (EC-PC) (Lewis et al. 2020; Zahlis et al. 2020).

##### Family Audiobook

An intervention involving audiobooks was described in 2 of the articles (Cuhls et al. 2021; Ateş et al. 2024). It aimed to help parents at the end of life in processing their emotions related to their illness and limited lifespan by creating personal audiobooks for their children, as part of palliative care. With the support of radio journalists, parents created professional recordings of their life stories, important memories, songs performed by themselves, selected music, readings, or other personally chosen content, intended as a lasting legacy for their children. Each audiobook was accompanied by a small booklet containing a table of contents and photos. The creative act of producing the audiobooks was intended to reduce psychological suffering and strengthen parents’ sense of meaning and closure. Although primarily psychosocial, the intervention also included biographical and existential elements, offering parents a space to reflect on their lives and experiences. Additionally, the audiobooks may serve as a concrete source of emotional support for the children, providing a valuable legacy during the grieving process.

Creating the audiobooks was associated with a sense of ease among the participating parents, who appreciated being able to leave behind a meaningful digital legacy, not only for their children but for the whole family (Cuhls et al. 2021; Ateş et al. 2024). Although some expressed concerns about how their children might react upon hearing the recordings, they hoped the audiobooks would offer comfort during future difficult times (Cuhls et al. 2021). While the recording process was described as emotionally challenging, parents still valued it as an opportunity to create a lasting bond with their children and would recommend the intervention to others. It was perceived to help them cope with their illness, express thoughts they found difficult to share otherwise, and promote more open dialogue within the family.

##### Enhancing Connections-Palliative Care (EC-PC)

Two articles reported findings related to the EC-PC program (Lewis et al. 2020; Zahlis et al. 2020). This was a telephone-delivered intervention, led by a trained nurse or patient educator, designed to help parents with incurable cancer manage their parental roles. By providing them with education, guidance, and strategies, it aimed to improve parents’ ability to support their children. The intervention included 5 sessions at 2-week interval, focusing on helping parents separate their own experiences from their child’s, manage emotions, enhance self-care, listen actively, and support their child. At-home assignments aimed to improve parents’ communication with their children, as well as strengthening their ability to handle their own with anxiety. While primarily psychoeducational, the intervention also included psychosocial elements encouraging emotional reflection and dialogue.

The EC-PC program was found to be feasible and well-received, with parents reporting increased self-efficacy in supporting their children emotionally, addressing their concerns and helping them cope with the situation (Lewis et al. 2020). A reduction in parental anxiety was seen, as well as improvements in parenting skills and children’s behavioral and emotional adjustment. Parents described EC-PC as providing tools that supported open communication and enabling deeper conversations with their children (Zahlis et al. 2020). It helped them become more aware of their children’s emotional needs, manage their own reactions during difficult discussions, and address topics they had previously avoided. EC-PC was also perceived to enhance parents’ awareness of the importance of active listening and creating space for their children to express themselves.

#### Family-based support intervention

Seven articles focused on the Family Talk Intervention (FTI), designed to support families living with parental life-threatening illness (Eklund et al. 2020, 2022a, 2022b, 2022c; Alvariza et al. 2021; Weber Falk et al. 2022; Bergersen et al. 2024).

##### Family Talk Intervention (FTI)

Findings related to the FTI were reported in 7 articles, each offering a different perspective (Eklund et al. 2020, 2022a, 2022b, 2022c; Alvariza et al. 2021; Weber Falk et al. 2022; Bergersen et al. 2024). The intervention aimed to improve communication within families where a parent is affected by a life-threatening illness, by encouraging open dialogue about the disease and its impact. FTI sought to help family members share experiences and feelings with each other, assisting families in identifying their strengths, and to support parents in recognizing and meeting their children’s needs. It comprised 6 structured meetings held 1–2 weeks apart, led by 2 FTI-trained interventionists, involving parents, children, or the whole family depending on the stage of the intervention. Although primarily psychosocial, FTI also included elements of psychoeducation, peer support, and practical assistance.

Quantitative measurements showed no significant changes in family communication before and after FTI, apart from improved satisfaction with conversations and an increased willingness to talk among ill parents (Weber Falk et al. 2022). However, no negative changes were reported (Eklund et al. 2020; Weber Falk et al. 2022). The intervention was positively received by both children and parents, with most indicating that it met their expectations and that they would recommend it to others (Alvariza et al. 2021; Eklund et al. 2022a). The majority stated that they felt understood by the interventionists and comfortable sharing their thoughts. Some children also reported increased knowledge about their parent’s illness (Eklund et al. 2020).

Children and parents described improved family communication and mutual understanding following the intervention (Eklund et al. 2020, 2022a, 2022c; Alvariza et al. 2021; Weber Falk et al. 2022; Bergersen et al. 2024). Parents found FTI helpful for initiating sensitive conversations, deepening dialogue, and becoming more attentive listeners (Alvariza et al. 2021; Eklund et al. 2022c; Weber Falk et al. 2022). Children appreciated feeling heard and experienced family discussions as more open (Eklund et al. 2020, 2022a). They valued the individual meeting without their parents (Eklund et al. 2022a), which parents also considered important for giving their child space to express themselves freely (Alvariza et al. 2021). While some children found participation emotionally challenging or noticed no major change in communication, most valued the supportive and safe environment FTI offered. Still, only a quarter of the children met the minimum level required by Article 12 of the UNCRC, where children’s views are meaningfully taken into account (Eklund et al. 2022b). To assess this, the Pathways to Participation model (Shier 2001) was applied. The model outlines 5 levels of participation, ranging from being listened to and supported in expressing views (levels 1–2), to having one’s views taken into account (level 3), and progressing toward shared decision-making and shared responsibility (levels 4–5).

The structured yet flexible format of FTI was generally appreciated by families, although several wished for earlier participation, more thorough preparation, additional meetings, or separate sessions to address sensitive topics (Alvariza et al. 2021; Eklund et al. 2022a). Children described how the intervention enhanced their understanding of the illness and helped them feel more prepared for future challenges, including potential medical emergencies (Eklund et al. 2020). Both ill parents and co-parents expressed a sense of security for the future following FTI, reassured that the family was better equipped to cope and support one another (Weber Falk et al. 2022).

The interventionists were described by children and parents as playing a central role in their overall experience, generally perceived as professional and as contributing to a sense of safety (Alvariza et al. 2021; Eklund et al. 2022a). The practical assistance they provided was also appreciated, for example, facilitating access to external support services for children, helping parents with organizing school meetings, obtaining aids, and navigating application processes (Eklund et al. 2020; Weber Falk et al. 2022). In some cases, the interventionists also enabled contact with other families in similar situations, and parents described this peer support as alleviating feelings of loneliness both during and after the intervention (Bergersen et al. 2024).

## Discussion

This review identified 15 articles describing 6 interventions aimed at supporting children and/or parents in families with a parent who had a life-threatening illness. Of these, 3 targeted children, 2 parents, and 1 the entire family. The interventions were primarily based on psychosocial, psychoeducational, and/or peer support but also incorporated additional supportive elements. Positive findings included improved family communication and mutual understanding, increased illness-related knowledge, enhanced coping, and reduced loneliness among children and parents. Gains in caregiving skills and parenting confidence were reported, and several interventions were considered feasible and acceptable. Although negative findings were few, some participants found their participation emotionally challenging. Overall, the interventions were perceived as helpful in navigating the difficult life situation, with several participants stating they would recommend them to others.

The limited number of included articles in this review – mostly small-scale pilot or feasibility studies covering few interventions – confirms the scarcity of research on support interventions for families facing parental life-threatening illness. It also reflects the previously reported knowledge gap in the context of fatal progressive neurological or neuro-oncological diseases. While the inclusion criteria were broadened to encompass various life-threatening illnesses, this review was primarily motivated by clinical and research interest in the neurological field. Although some interventions targeted a broader range of diseases, most were either designed specifically for cancer or included predominantly cancer-affected participants, with limited representation of other diagnoses. With only 3 articles describing 2 interventions focusing exclusively on fatal progressive neurological diseases (Kavanaugh et al. 2017, 2018, 2020a), the urgent need for more tailored support within the neurological care context is clearly highlighted. While all life-threatening illnesses can have a profound impact on families, those facing progressive neurological or neuro-oncological diseases may encounter distinct and complex challenges. Typical symptoms, such as gradually increasing physical impairment and, in some diagnoses, difficulties with breathing, speaking or swallowing (Masrori and Van Damme 2020; Stoker et al. 2022; Schaff and Mellinghoff 2023), can complicate parents’ ability to care for their children and express physical affection. The fear of choking and dying from sudden respiratory failure is also a major source of worry among both children and parents (Stoker et al. 2022; Malmström et al. 2025), reflecting a lack of knowledge about the disease. Additionally, cognitive decline and behavioral changes, which may occur (Masrori and Van Damme 2020; Pender et al. 2020; Stoker et al. 2022; Schaff and Mellinghoff 2023), can impair parents’ capacity to be mentally present and emotionally supportive. These challenges may become even more complicated if speech is impaired and communication limited. Brain tumors have also been associated with increased externalized aggression, adding to families’ emotional strain and instability (Arifin et al. 2014; Boele et al. 2015).

The overall positive experiences across the interventions underline the importance of providing families with support, rather than prioritizing a specific type, as any formal support may be preferable to leaving families to navigate the situation on their own (Malmström et al. 2024, 2025; Billaud Feragen et al. 2025; Cooper et al. 2025). Simultaneously, as needs often vary – both between and within families – different forms of support remain essential. While there may be uncertainty about where to turn for support, families may also have limited time and energy to search for it (Malmström et al. 2023, 2025). This further underscores the importance of early and proactive support, to avoid placing the burden on families to initiate help-seeking themselves before the situation may escalate. Given the gradual progression, support from the point of diagnosis with ongoing follow-up is essential throughout the disease trajectory, with palliative care playing a central role – shown to be both feasible and valued by ill parents and their families (Fahrner-Scott et al. 2022; Zwicker et al. 2023).

While it is important to tailor support to the neurological context, several components of the interventions identified in this review may still hold value. Support to improve communication has previously been requested, including family therapy to foster dialogue and stronger relationships, as well as individual sessions (Sommers-Spijkerman et al. 2022; Malmström et al. 2023). The structure of FTI was described as flexible to the families’ needs (Alvariza et al. 2021), which is important given that the ill parent’s condition may deteriorate rapidly and create new everyday challenges. As symptoms may also impair verbal interaction cognitively or physically, families should be supported in identifying alternative strategies for maintaining communication. With the audiobook intervention in mind (Cuhls et al. 2021; Ateş et al. 2024), guidance and tools for recording voice messages before speech becomes severely impaired may be particularly valuable.

Research highlights the need for families facing fatal progressive neurological disease to connect with others in similar situations (Kavanaugh et al. 2015, 2021; Malmström et al. 2023). As shown in the results, peer camps can help children find a sense of belonging and reduce feelings of isolation (Kavanaugh et al. 2017), while also offering temporary respite from the illness context – an essential need for their well-being (Malmström et al. 2023). However, various meeting formats can be valuable, including support groups organized by healthcare, social services, or other organizations. Such support may come not only from professionals but also from civil society and community-based initiatives, reflecting a shared and ideally early responsibility, consistent with a public health palliative care perspective (Sallnow et al. 2022). In addition to addressing practical and emotional needs, families also require support to process existential dimensions of the situation and the uncertainty it brings, including gaps in information about the disease and its progression.

Families facing a fatal progressive neurological or neuro-oncological illness often express frustration and worry due to a lack of clear information (Loughan et al. 2022; Malmström et al. 2023, 2024, 2025; Cooper et al. 2025). This must be carefully considered when adapting psychoeducational interventions to the neurological context and underscores the importance of specialist expertise among those delivering the support. Involving children and ensuring their access to professionals is essential for promoting understanding (Malmström et al. 2023), as illustrated by the expert-led training day intervention, which was considered valuable and helped increase children’s feelings of preparedness (Kavanaugh et al. 2020a, 2018). This highlights the need for educational support for caregiving children, as well as adequate practical support for families to prevent children from becoming overburdened. Research shows that children may compensate for gaps in such support by taking on extensive responsibilities (Bergersen et al. 2022; Malmström et al. 2024, 2025; Cooper et al. 2025). It is therefore crucial to ensure that children are not placed in a position of disproportionate responsibility – especially in contexts with limited home care services.

Digital support has been requested by families affected by fatal progressive neurological illness to improve accessibility when in-person sessions are difficult due to physical limitations or caregiving demands (Malmström et al. 2023, 2024). Although this review did not identify any digital support, the telephone-based intervention may have helped some of the ill parents to overcome some of these participation barriers (Cuhls et al. 2021; Ateş et al. 2024). Digital support may be particularly relevant for children, as it offers a familiar setting while also improving access for those who may remain at home due to extensive responsibilities and fear of leaving their ill parent (Malmström et al. 2024).

Previous research has highlighted the need for a holistic approach from healthcare when encountering a life-threateningly ill patient who is also a parent (Malmström et al. 2023, 2025; Billaud Feragen et al. 2025). Requests from both children and parents for mandatory family meetings at the time of diagnosis reflect the urgency of establishing such meetings as a routine part of care (Malmström et al. 2023). While a more proactive approach to supporting the family as a whole may be essential to overall family health, attention should also be given to individual support. Children may require professional help to understand and process their emotions, and to cope with the consequences of the illness (Malmström et al. 2023, 2024). Given children’s appreciation of the FTI’s individual meetings (Eklund et al. 2022a), along with previous findings indicating that they often refrain from sharing difficult thoughts to avoid burdening their parents (Malmström et al. 2024), it is important that they have access to a safe and trusting environment in which to express their emotions. However, as children’s views were not always fully taken into account within FTI (Eklund et al. 2022b), it remains essential to ensure that their perspectives are genuinely heard and valued. In some cases, alternative methods of communication may facilitate emotional expression rather than verbal interaction. This highlights the relevance of the art-therapeutic elements included in MKWF, although the structure and implementation of that intervention were unfortunately described in limited detail (Holland et al. 2018). As symptoms progress and increasingly affect parenthood, parental support may also become necessary (Billaud Feragen et al. 2025; Malmström et al. 2025). Both ill parents and co-parents may need help to cope with the illness and their own emotional reactions, as well as to better understand and support their children. Additionally, ill parents should be provided with guidance on how to maintain a stable and supportive parental presence despite physical and cognitive decline. All in all, it is essential to recognize families in their relational and everyday context, and to draw on both formal and informal support in responding to their shared and individual needs (Sallnow et al. 2022).

Although most included studies were small-scale feasibility or pilot studies, limiting conclusions about the interventions’ effectiveness, they still provide valuable insights for future support development. Several included components found meaningful by participants, particularly in relation to communication and emotional coping. Study quality and the clarity of intervention descriptions, as assessed by MMAT (Hong et al. 2018), varied. While some studies demonstrated strong coherence between data collection, analysis, and interpretation, others lacked transparency or clear methodological rationale. Some interventions were supported by multiple publications using both qualitative and quantitative methods, suggesting a relatively robust evidence base – for instance, FTI, which was examined in several high-quality-rated studies. Others were only described in a single publication, making broader relevance harder to assess. Although studies were not excluded based on quality, the appraisal highlights both methodological strengths and gaps. Future research should use more rigorous designs that combine outcome evaluation with exploration of lived experience to develop interventions that are effective, feasible, acceptable, and adaptable to different contexts.

In conclusion, this review highlights the limited availability of support interventions for families facing parental life-threatening illnesses, as well as in the context of fatal progressive neurological diseases. It underscores the need to develop interventions specifically tailored for these families, while existing approaches may still offer valuable guidance. There is a pressing need for early, proactive, and accessible support that addresses both individual and family needs – support that is holistic, adaptable to disease progression, and includes ongoing follow-up, reflecting core elements of high-quality palliative care.

## Supporting information

10.1017/S1478951526101837.sm001 Malmström et al. supplementary material Malmström et al. supplementary material

## Supplementary material

The supplementary material for this article can be found at https://doi.org/10.1017/S1478951526101837.
