# Supplementary material for: Support interventions for families facing parental life-threatening illness – A scoping review
Source: Palliat Support Care. 2026 Apr 14;24:e78. doi: 10.1017/S1478951526101837 (PMC13166525; doi:10.1017/S1478951526101837)
Supplement: Malmström et al. supplementary material [file S1478951526101837sup001.docx]

**Supplementary Materials**

Supplementary File 1. Overview of searches in the databases

| **PubMed** | | | |
| --- | --- | --- | --- |
| Interface: pubmed.gov  Date of Search: 2023-11-10^1^  Number of hits: 2 742 | | Field labels   - [mh] or [mesh] = exploded MeSH term - [tiab] = title, abstract - * = truncation of word for alternate endings - NB If truncating a phrase – the truncated term must be the last word in the phrase. | |
| **#** | **Searches** | | **Results** |
| #1 | support*[tiab] OR social support[mesh] OR family support[mesh] OR intervention[tiab] OR program*[tiab] OR "model"[tiab] | | 5 890 949 |
| #2 | child*[tiab] OR child[mesh] OR Adolescent[mesh] OR adolescent*[tiab] OR teenager*[tiab] OR youth*[tiab] OR young*[tiab] OR Minors[mesh] OR minor*[tiab] | | 4 721 159 |
| #3 | Famil*[tiab] OR sibling*[tiab] OR sister*[tiab] OR brother*[tiab] OR mother*[tiab] OR father*[tiab] OR family[mesh] OR relative*[tiab] OR parent*[tiab] OR siblings[mesh] OR mothers[mesh] OR fathers[mesh] OR parents[mesh] | | 3 618 177 |
| #4 | Hospice and palliative care nursing[mesh] OR palliative medicine[mesh] OR palliative care[mesh] OR palliative care*[tiab] OR terminal care*[tiab] OR terminal care[mesh] OR end-of-life care[tiab] OR life-threatening disease[tiab] OR life-threatening illness[tiab] OR progressive neurological disease[tiab] OR progressive neurological illness[tiab] OR Amyotrophic Lateral Sclerosis[mesh] OR Amyotrophic Lateral Scleros*[tiab] OR Gehrig Disease*[tiab] OR Guam Disease*[tiab] OR ALS[tiab] OR MND[tiab] OR Huntington Disease[mesh] OR Huntington*[tiab] OR Parkinson Disease[mesh] OR Parkinson*[tiab] OR Brain Neoplasms[mesh] OR brain neoplasm*[tiab] OR brain tumour*[tiab] OR brain tumor*[tiab] OR brain cancer[tiab] OR incurable cancer[tiab] OR terminal cancer[tiab] | | 534 425 |
| #5 | #1 AND #2 AND #3 AND #4 | | 4 830 |
| #6 | Limit years 2013-2023 | | 2 742 |
| **PsycInfo** | | | |
| Interface: ProQuest  Date of Search: 2023-11-10^1^  Number of hits: 1 105 | | Field labels   - TIAB = title, abstract - TIABSU = title, abstract, subject - NEAR/x = within x words, regardless of order - * = truncation of word for alternate endings | |
| **#** | **Searches** | | **Results** |
| #1 | MAINSUBJECT.EXACT.EXPLODE("Social Support") OR MAINSUBJECT.EXACT.EXPLODE("Emotional Support") OR TIAB(support*) OR MAINSUBJECT.EXACT.EXPLODE("Intervention") OR TIAB(intervention*) OR TIAB(program*) OR TIAB(model*) | | 1 891 665 |
| #2 | TIAB(child*) OR TIAB(adolescen*) OR TIAB(teenager*) OR TIAB(youth*) OR TIAB(young*) OR TIAB(minor*) | | 1 135 653 |
| #3 | MAINSUBJECT.EXACT.EXPLODE("Family") OR TIAB(famil*) OR MAINSUBJECT.EXACT.EXPLODE("Siblings") OR TIAB(sibling*) OR MAINSUBJECT.EXACT.EXPLODE("Parents") OR TIAB(parent*) OR TIAB(relative*) OR MAINSUBJECT.EXACT.EXPLODE("Kinship") OR TIAB(kinship*) OR MAINSUBJECT.EXACT.EXPLODE("Sisters") OR TIAB(sister*) OR MAINSUBJECT.EXACT.EXPLODE("Brothers") OR TIAB(brother*) OR MAINSUBJECT.EXACT.EXPLODE("Mothers") OR TIAB(mother*) OR MAINSUBJECT.EXACT.EXPLODE("Fathers") OR TIAB(father*) | | 1 021 077 |
| #4 | MAINSUBJECT.EXACT.EXPLODE("Brain Neoplasms") OR TIAB(“brain neoplasm*”) OR TIAB(“brain tumour*”) OR TIAB(“brain tumor*”) OR TIAB(“brain cancer*”) OR MAINSUBJECT.EXACT.EXPLODE("Palliative Care") OR TIAB(“palliative care”) OR TIAB(“palliative treatment”) OR TIAB(“terminal care”) OR TIAB(“end-of-life care”) OR TIAB("life-threatening disease") OR TIAB("life-threatening illness") OR TIAB("progressive neurological disease") OR TIAB("progressive neurological illness") OR MAINSUBJECT.EXACT.EXPLODE("Amyotrophic Lateral Sclerosis") OR TIAB("Amyotrophic Lateral Scleros*") OR TIAB("Gehrig Disease*") OR TIAB("Guam Disease*") OR TIAB(ALS) OR TIAB(MND) OR MAINSUBJECT.EXACT.EXPLODE("Huntingtons Disease") OR TIAB(Huntington*) OR MAINSUBJECT.EXACT.EXPLODE("Parkinson's Disease") OR TIAB(Parkinson*) OR TIAB("incurable cancer") OR TIAB("terminal cancer") | | 83 788 |
| #5 | #1 AND #2 AND #3 AND #4 | | 1 765 |
| #6 | Limit years 2013-2023, Peer review | | 1 105 |
| **Cinahl** | | | |
| Interface: Ebsco  Date of Search: 2023-11-10^1^  Number of hits: 489 | | Field labels   - MH+ = exploded Cinahl Heading - MH = non exploded Cinahl Heading - TI = title - AB = abstract - Nx = within x words, regardless of order - * = truncation of word for alternate endings | |
| **#** | **Searches** | | **Results** |
| #1 | (MH "Support, Psychosocial+") OR (MH "Support, Social+") OR (MH "Family Support") OR TI support OR AB support OR TI social care OR AB social care TI intervention OR AB intervention OR TI program* OR AB program* OR TI model* OR AB model* | | 439 582 |
| #2 | (TI child* OR AB child*) OR (MH child+) OR (MH Adolescence+) OR (TI adolescen* OR AB adolescen*) OR (TI teenager* OR AB teenager*) OR (TI youth* OR AB youth*) OR (TI young* OR AB young*) OR (MH Minors (Legal)+) OR (TI minor* OR AB minor*) | | 388 050 |
| #3 | (TI Famil* OR AB Famil*) OR (TI sibling* OR AB sibling*) OR (TI sister* OR AB sister*) OR (TI brother* OR AB brother*) OR (TI mother* OR AB mother*) OR (TI father* OR AB father*) OR (MH family+) OR (TI relative* OR AB relative*) OR (TI parent* OR AB parent*) OR (MH siblings+) OR (MH mothers+) OR (MH fathers+) OR (MH parents+) | | 221 766 |
| #4 | (MH "palliative care nursing+") OR (MH "palliative medicine+") OR (MH "palliative care+") OR (TI "palliative care*" OR AB "palliative care*") OR (TI "terminal care*" OR AB "terminal care*") OR (MH "terminal care+") OR (TI "end-of-life care" OR AB "end-of-life care") OR (TI "life-threatening disease" OR AB "life-threatening disease") OR (TI "life-threatening illness" OR AB "life-threatening illness") OR (MH "Critical Illness") OR (TI "progressive neurological disease" OR AB "progressive neurological disease") OR (TI "progressive neurological illness" OR AB "progressive neurological illness") OR (MH "Amyotrophic Lateral Sclerosis+") OR (TI "Amyotrophic Lateral Scleros*" OR AB "Amyotrophic Lateral Scleros*") OR (TI "Gehrig Disease*" OR AB "Gehrig Disease*") OR (TI "Guam Disease*" OR AB "Guam Disease*") OR (TI ALS OR AB ALS) OR (TI MND OR AB MND) OR (MH "Huntington's Disease +") OR (TI Huntington* OR AB Huntington*) OR (MH "Parkinson Disease+") OR (TI Parkinson* OR AB Parkinson*) OR (MH "Brain Neoplasms+") OR (TI "brain neoplasm*" OR AB "brain neoplasm*") OR (TI "brain tumour*" OR AB "brain tumour*") OR (TI "brain tumor*" OR AB "brain tumor*") OR (TI "brain cancer" OR AB "brain cancer") OR (TI "incurable cancer" OR AB "incurable cancer") OR (TI "terminal cancer" OR AB "terminal cancer") | | 45 555 |
| #5 | #1 AND #2 AND #3 AND #4 | | 790 |
| #6 | Limit years 2013-2023, Peer review | | 489 |
| **Web of Science Core Collection** | | | |
| Interface: Clarivate Analytics  Date of Search: 2023-11-10^1^  Number of hits: 2 699 | | Field labels   - TS/Topic = title, abstract, author keywords and Keywords Plus - NEAR/x = within x words, regardless of order - * = truncation of word for alternate endings | |
| **#** | **Searches** | | **Results** |
| #1 | (support* OR intervention* OR program* OR model*) Topic | | 14 510 636 |
| #2 | (child* OR adolescen* OR teenager* OR youth* OR young* OR minor*) Topic | | 3 851 339 |
| #3 | (Famil* OR sibling* OR sister* OR brother* OR mother* OR father* OR relative* OR parent*) Topic | | 5 440 463 |
| #4 | ("palliative medicine*" OR "palliative care*" OR "terminal care*" OR "end-of-life care*" OR "life-threatening disease*" OR "life-threatening illness*" OR "progressive neurological disease*" OR "progressive neurological illness*" OR "Amyotrophic Lateral Scleros*" OR "Gehrig Disease*" OR "Guam Disease*" OR ALS OR MND OR Huntington* OR Parkinson* OR "brain neoplasm*" OR "brain tumour*" OR "brain tumor*" OR "brain cancer" OR "incurable cancer" OR "terminal cancer") Topic | | 439 922 |
| #5 | #1 AND #2 AND #3 AND #4 | | 4 689 |
| #6 | Limit years 2013-2023, Article | | 2 699 |

^1^ *An updated search was conducted on 12-02-25 using the same search strategy, databases, and search blocks as described above. It resulted in 710 articles after duplicate removal.*

Supplementary File 2. Quality assessment of included studies using the Mixed Methods Appraisal Tool (MMAT), 2018 Version

| **Category of study designs** | **Methodological quality criteria** | **Response**  **(Yes / No / Can't tell)** | **Comments** |
| --- | --- | --- | --- |
| **1. Qualitative** | | | |
| Cuhls et al., 2021 | **Screening questions (for all types):** |  |  |
|  | S1. Are there clear research questions? | Yes | The research question is clearly stated in the aim, which is to evaluate an audiobook intervention for terminally ill parents with young children. |
|  | S2. Do the collected data allow to address the research questions? | Yes | The data collected through semi-structured interviews are appropriate to address the research question. |
|  | **Criteria of chosen category:** |  |  |
|  | 1.1. Is the qualitative approach appropriate to answer the research question? | Yes | The qualitative approach is appropriate to answer the research question, as it focuses on exploring personal experiences. |
|  | 1.2. Are the qualitative data collection methods adequate to address the research question? | Yes | Data collection through semi-structured interviews before and after participation is adequate to address the research question. |
|  | 1.3. Are the findings adequately derived from the data? | Yes | The findings are systematically derived through content analysis, using both deductive coding based on interview questions and inductive review of the entire material. |
|  | 1.4. Is the interpretation of results sufficiently substantiated by data? | Yes | The interpretation of results is substantiated by illustrative quotes that support the categories. |
|  | 1.5. Is there coherence between qualitative data sources, collection, analysis and interpretation? | Yes | The qualitative data collection, analysis and interpretation are coherent throughout the study. |
|  | **Number of criteria met:** | **5/5 = Good quality** |  |
| Zahlis et al. 2020 | **Screening questions (for all types):** |  |  |
|  | S1. Are there clear research questions? | Yes | The research question is clearly stated in the aim, which is to describe parents’ attributed gains from participating in a fully manualized psycho-educational program designed to enhance their interactional skills, competencies, and confidence in communicating with their child about the parent’s incurable cancer. |
|  | S2. Do the collected data allow to address the research questions? | Yes | The data collected through open-ended questions via telephone allow the research question to be addressed. |
|  | **Criteria of chosen category:** |  |  |
|  | 1.1. Is the qualitative approach appropriate to answer the research question? | Yes | The qualitative approach is appropriate to answer the research question, as it focuses on personal reflections and perceived gains from the intervention. |
|  | 1.2. Are the qualitative data collection methods adequate to address the research question? | No | Data were collected by the same person who carried out the intervention, which potentially involves bias. |
|  | 1.3. Are the findings adequately derived from the data? | Yes | The findings are adequately derived from the data using a structured inductive content analysis, including coding, categorization, and constant comparative analysis. |
|  | 1.4. Is the interpretation of results sufficiently substantiated by data? | Yes | The interpretation of results is based on categories developed from participants’ responses and supported by illustrative quotes. |
|  | 1.5. Is there coherence between qualitative data sources, collection, analysis and interpretation? | Yes | The qualitative data collection, analysis and interpretation are coherent throughout the study. |
|  | **Number of criteria met:** | **4/5 = Good quality** |  |
| Eklund et al., 2022a | **Screening questions (for all types):** |  |  |
|  | S1. Are there clear research questions? | Yes | The research question is clearly stated in the aim, which is to explore the child’s active participation in the Family Talk Intervention, in accordance with Article 12 of the United Nations Convention on the Rights of the Child, when having a parent cared for in palliative care. |
|  | S2. Do the collected data allow to address the research questions? | Yes | Data collected through field notes written during the intervention sessions provide relevant data to address the research question. |
|  | **Criteria of chosen category:** |  |  |
|  | 1.1. Is the qualitative approach appropriate to answer the research question? | Yes | The qualitative approach is appropriate for exploring children’s participation within the context of the intervention. |
|  | 1.2. Are the qualitative data collection methods adequate to address the research question? | Yes | Field notes written during the intervention by the interventionists are appropriate for capturing children’s participation within the context of the intervention, making the data collection method adequate to address the research question. |
|  | 1.3. Are the findings adequately derived from the data? | Yes | Findings are adequately derived from the data through a systematic, interpretive analysis, supported by memos and a guiding framework. |
|  | 1.4. Is the interpretation of results sufficiently substantiated by data? | Yes | The interpretation is supported by a theory-guided framework of participation that shows how children’s views were taken into account during the intervention, also reflected in illustrative quotes. |
|  | 1.5. Is there coherence between qualitative data sources, collection, analysis and interpretation? | Yes | The qualitative data collection, analysis and interpretation are coherent throughout the study. |
|  | **Number of criteria met:** | **5/5 = Good quality** |  |
| Bergersen et al., 2024 | **Screening questions (for all types):** |  |  |
|  | S1. Are there clear research questions? | Yes | The research questions are clearly stated in the aim, which is to describe the parents’ perceptions of the timing and length of FTI in relation to the illness trajectory, to explore what activities learnt by the FTI still were practiced in the long-term and what content of FTI was perceived as most valuable to coping in the long-term. |
|  | S2. Do the collected data allow to address the research questions? | Yes | The semi-structured interviews conducted 4-5 years after participation in FTI provide relevant data to address the research questions. |
|  | **Criteria of chosen category:** |  |  |
|  | 1.1. Is the qualitative approach appropriate to answer the research question? | Yes | The qualitative approach is appropriate for exploring long-term personal experiences. |
|  | 1.2. Are the qualitative data collection methods adequate to address the research question? | Yes | Semi-structured interviews are a suitable method for capturing personal experiences. |
|  | 1.3. Are the findings adequately derived from the data? | Yes | The findings are adequately derived through systematic phenomenographic analysis. |
|  | 1.4. Is the interpretation of results sufficiently substantiated by data? | Yes | Interpretations are substantiated by data, with quotes to support the identified categories. |
|  | 1.5. Is there coherence between qualitative data sources, collection, analysis and interpretation? | Yes | The qualitative data collection, analysis and interpretation are coherent throughout the study. |
|  | **Number of criteria met:** | **5/5 = Good quality** |  |
| **3. Quantitative non-randomized** | | | |
| Lewis et al., 2020 | **Screening questions (for all types):** |  |  |
|  | S1. Are there clear research questions? | Yes | The research questions are clearly stated in the aim, which is to report the feasibility and short-term impact of the EC-PC program. |
|  | S2. Do the collected data allow to address the research questions? | Yes | The data collected using pre/post questionnaires are appropriate to address the research question. |
|  | **Criteria of chosen category:** |  |  |
|  | 3.1. Are the participants representative of the target population? | Yes | The participants match the intended population for the intervention. |
|  | 3.2. Are measurements appropriate regarding both the outcome and intervention (or exposure)? | Yes | Valid and appropriate instruments were used to measure outcomes in line with the study aim, including CES-D, STAI, and Parenting Confidence. |
|  | 3.3. Are there complete outcome data? | Yes | Outcome data are considered complete with missing data adequately addressed. |
|  | 3.4. Are the confounders accounted for in the design and analysis? | Yes | Between-group analyses were adjusted for covariates, accounting for potential confounding factors. |
|  | 3.5. During the study period, is the intervention administered (or exposure occurred) as intended? | Yes | The intervention was delivered as intended. |
|  | **Number of criteria met:** | **5/5 = Good quality** |  |
| **4. Quantitative descriptive** | | | |
| Ateş et al., 2024 | **Screening questions (for all types):** |  |  |
|  | S1. Are there clear research questions? | Yes | The research question is clearly stated in the aim, which is to examine how terminally ill parents and those close to them perceive the audiobook and how satisfied they are with it. |
|  | S2. Do the collected data allow to address the research questions? | Yes | The data collected through online questionnaires are appropriate to address the research question. |
|  | **Criteria of chosen category:** |  |  |
|  | 4.1. Is the sampling strategy relevant to address the research question? | Yes | The sampling strategy targets individuals with direct experience of the audiobook intervention, which is relevant to address the research question. |
|  | 4.2. Is the sample representative of the target population? | No | The authors acknowledge that a non-representative snowball sample was used, with self-selection and no controlled respondent list. |
|  | 4.3. Are the measurements appropriate? | Yes | The questionnaire includes structured questions, and the examples provided suggest relevance to the study aim. |
|  | 4.4. Is the risk of nonresponse bias low? | Yes | The high response rate suggests a low risk of nonresponse bias, although there is some uncertainty due to snowball sampling and the lack of a controlled respondent list. |
|  | 4.5. Is the statistical analysis appropriate to answer the research question? | Yes | Descriptive statistics and bivariate analysis are appropriate to answer the research question. |
|  | **Number of criteria met:** | **4/5 = Good quality** |  |
| **5. Mixed methods** | | | |
| Kavanaugh et al., 2018 | **Screening questions (for all types):** |  |  |
|  | S1. Are there clear research questions? | Yes | The research question is clearly stated in the aim, which is to assess the feasibility of a multidisciplinary young caregiver group training protocol for children and youths who provide care to a family member with ALS. |
|  | S2. Do the collected data allow to address the research questions? | Yes | The use of both pre/post questionnaires and check-in questionnaires/discussions between the modules provides relevant data to address the research question. |
|  | **Criteria of chosen category:** |  |  |
|  | 5.1. Is there an adequate rationale for using a mixed methods design to address the research question? | No | No reason for conducting a mixed methods study is stated. |
|  | 5.2. Are the different components of the study effectively integrated to answer the research question? | Yes | Some integration of the different components occurs in both the results and the discussion, where qualitative reflections from group discussions or responses on open-ended questions are used to help illustrate the quantitative parts. |
|  | 5.3. Are the outputs of the integration of qualitative and quantitative components adequately interpreted? | Yes | To some extent, qualitative reflections are used to support interpretation of quantitative findings in the discussion. |
|  | 5.4. Are divergences and inconsistencies between quantitative and qualitative results adequately addressed? | Yes | No divergences or inconsistencies were identified. |
|  | 5.5. Do the different components of the study adhere to the quality criteria of each tradition of the methods involved? | No | Both method components lack a described analysis method, limiting the ability to assess quality. |
|  | **Number of criteria met:** | **3/5 = Medium quality** |  |
| Kavanaugh et al., 2020 | **Screening questions (for all types):** |  |  |
|  | S1. Are there clear research questions? | Yes | The research questions are clearly stated in the aim, which is to explore changes in youth caregivers’ self-efficacy and self-care awareness after YCare participation. |
|  | S2. Do the collected data allow to address the research questions? | Yes | The data collected through quantitative surveys and brief module reflections are appropriate to answer the research questions. |
|  | **Criteria of chosen category:** |  |  |
|  | 5.1. Is there an adequate rationale for using a mixed methods design to address the research question? | No | No reason for conducting a mixed methods study is stated. |
|  | 5.2. Are the different components of the study effectively integrated to answer the research question? | Yes | Qualitative and quantitative findings are reported separately in the results, but some integration occurs in the discussion. |
|  | 5.3. Are the outputs of the integration of qualitative and quantitative components adequately interpreted? | Yes | To some extent, qualitative and quantitative findings are interpreted in relation to each other in the discussion. |
|  | 5.4. Are divergences and inconsistencies between quantitative and qualitative results adequately addressed? | Yes | No divergences or inconsistencies were identified. |
|  | 5.5. Do the different components of the study adhere to the quality criteria of each tradition of the methods involved? | No | The qualitative analysis method is not described, and the reporting of the quantitative analysis is limited. |
|  | **Number of criteria met:** | **3/5 = Medium quality** |  |
| Kavanaugh et al., 2017 | **Screening questions (for all types):** |  |  |
|  | S1. Are there clear research questions? | Yes | The research questions are clearly stated in the aim, which is to evaluate the effects of the camp, primarily by examining how the camp affected the youths’ feelings of isolation and their ability to receive support from others in similar situations. |
|  | S2. Do the collected data allow to address the research questions? | Yes | The data collected through repeated surveys and open-ended questions are appropriate for addressing the research question regarding the camp’s impact on youth participants. However, the very small sample size may limit the ability to fully answer the research question. |
|  | **Criteria of chosen category:** |  |  |
|  | 5.1. Is there an adequate rationale for using a mixed methods design to address the research question? | Yes | The reason for conducting a mixed methods study is explained. |
|  | 5.2. Are the different components of the study effectively integrated to answer the research question? | Yes | Integration of qualitative and quantitative findings occurs both in the results and the discussion. |
|  | 5.3. Are the outputs of the integration of qualitative and quantitative components adequately interpreted? | Yes | Qualitative and quantitative findings are interpreted in relation to each other. |
|  | 5.4. Are divergences and inconsistencies between quantitative and qualitative results adequately addressed? | Yes | No divergences or inconsistencies were identified. |
|  | 5.5. Do the different components of the study adhere to the quality criteria of each tradition of the methods involved? | Yes | Both the qualitative and quantitative components are well described and meet their quality criteria. |
|  | **Number of criteria met:** | **5/5 = Good quality** |  |
| Holland et al., 2018 | **Screening questions (for all types):** |  |  |
|  | S1. Are there clear research questions? | Yes | The research question is clearly stated in the aim, which is to develop a toolbox to deepen children’s understanding of parental cancer and support parent-child communication. |
|  | S2. Do the collected data allow to address the research questions? | Yes | The data collected through quantitative surveys and semi-structured qualitative interviews are appropriate to answer the research questions. |
|  | **Criteria of chosen category:** |  |  |
|  | 5.1. Is there an adequate rationale for using a mixed methods design to address the research question? | No | No reason for conducting a mixed methods study is stated. |
|  | 5.2. Are the different components of the study effectively integrated to answer the research question? | Can’t tell | Integration of quantitative and qualitative data is unclear, as components are mostly presented separately. |
|  | 5.3. Are the outputs of the integration of qualitative and quantitative components adequately interpreted? | No | There is no clear interpretation that combines both types of data. |
|  | 5.4. Are divergences and inconsistencies between quantitative and qualitative results adequately addressed? | Yes | No divergences or inconsistencies were identified. |
|  | 5.5. Do the different components of the study adhere to the quality criteria of each tradition of the methods involved? | No | The qualitative analysis (cross-case thematic) is described, but there is no clear description of how the quantitative data were analyzed apart from reporting descriptive figures. |
|  | **Number of criteria met:** | **1/5 = Low quality** |  |
| Alvariza et al., 2020 | **Screening questions (for all types):** |  |  |
|  | S1. Are there clear research questions? | Yes | The research question is clearly stated in the aim, which is to evaluate the feasibility of FTI in terms of acceptability from the perspective of parents in families where a parent suffers from a life-threatening illness and receives specialized palliative home care. |
|  | S2. Do the collected data allow to address the research questions? | Yes | Study-specific questionnaires and interviews collected post-intervention provide relevant data to address the research question. |
|  | **Criteria of chosen category:** |  |  |
|  | 5.1. Is there an adequate rationale for using a mixed methods design to address the research question? | Yes | The reason for conducting a mixed methods study is explained. |
|  | 5.2. Are the different components of the study effectively integrated to answer the research question? | Yes | Integration of qualitative and quantitative findings is not found in the results but occurs in the discussion. |
|  | 5.3. Are the outputs of the integration of qualitative and quantitative components adequately interpreted? | Yes | The interpretation includes both qualitative and quantitative findings, considered together in the discussion to form an overall understanding. |
|  | 5.4. Are divergences and inconsistencies between quantitative and qualitative results adequately addressed? | Yes | No divergences or inconsistencies were identified. |
|  | 5.5. Do the different components of the study adhere to the quality criteria of each tradition of the methods involved? | Yes | Both the qualitative and quantitative components are well described and meet their quality criteria. |
|  | **Number of criteria met:** | **5/5 = Good quality** |  |
| Eklund et al., 2020 | **Screening questions (for all types):** |  |  |
|  | S1. Are there clear research questions? | Yes | The research question is clearly stated in the aim, which is to explore the potential effects of FTI from the perspectives of minor children whose parent is cared for in specialized palliative home care. |
|  | S2. Do the collected data allow to address the research questions? | Yes | Study-specific questionnaires and interviews collected post-intervention from children provide relevant data to address the research question. |
|  | **Criteria of chosen category:** |  |  |
|  | 5.1. Is there an adequate rationale for using a mixed methods design to address the research question? | Yes | The reason for conducting a mixed methods study is explained. |
|  | 5.2. Are the different components of the study effectively integrated to answer the research question? | Yes | Integration of qualitative and quantitative findings occurs both in the results and the discussion. |
|  | 5.3. Are the outputs of the integration of qualitative and quantitative components adequately interpreted? | Yes | Qualitative and quantitative findings are considered together to support an overall interpretation of the children’s experiences with the intervention. |
|  | 5.4. Are divergences and inconsistencies between quantitative and qualitative results adequately addressed? | Yes | No divergences or inconsistencies were identified. |
|  | 5.5. Do the different components of the study adhere to the quality criteria of each tradition of the methods involved? | Yes | Descriptions of both qualitative and quantitative methods are detailed and appropriate. |
|  | **Number of criteria met:** | **5/5 = Good quality** |  |
| Weber Falk et al., 2021 | **Screening questions (for all types):** |  |  |
|  | S1. Are there clear research questions? | Yes | The research question is clearly stated in the aim, which is to explore potential effects of FTI in specialized palliative home care from the ill parent’s and co-parent’s perspectives. |
|  | S2. Do the collected data allow to address the research questions? | Yes | Questionnaires and interviews collected post-intervention from parents provide relevant data to address the research question. |
|  | **Criteria of chosen category:** |  |  |
|  | 5.1. Is there an adequate rationale for using a mixed methods design to address the research question? | Yes | The reason for conducting a mixed methods study is motivated. |
|  | 5.2. Are the different components of the study effectively integrated to answer the research question? | Yes | Integration of qualitative and quantitative findings is clear in both results and discussion. |
|  | 5.3. Are the outputs of the integration of qualitative and quantitative components adequately interpreted? | Yes | The interpretation includes connections between qualitative and quantitative findings, as the participants’ own descriptions help explain the results from the questionnaires. |
|  | 5.4. Are divergences and inconsistencies between quantitative and qualitative results adequately addressed? | Yes | Divergences are clearly explained. |
|  | 5.5. Do the different components of the study adhere to the quality criteria of each tradition of the methods involved? | Yes | Both the qualitative and quantitative components are well described and meet their quality criteria. |
|  | **Number of criteria met:** | **5/5 = Good quality** |  |
| Eklund et al., 2022b | **Screening questions (for all types):** |  |  |
|  | S1. Are there clear research questions? | Yes | The research questions are clearly stated in the aim, which is to explore the feasibility of the family talk intervention (FTI) and its acceptability to dependent children when a parent is cared for in palliative home care. |
|  | S2. Do the collected data allow to address the research questions? | Yes | Study-specific questionnaires and interviews collected post-intervention from children provide relevant data to address the research question. |
|  | **Criteria of chosen category:** |  |  |
|  | 5.1. Is there an adequate rationale for using a mixed methods design to address the research question? | Yes | The reasons for conducting a mixed methods study are explained. |
|  | 5.2. Are the different components of the study effectively integrated to answer the research question? | Yes | Both qualitative and quantitative data are brought together in results and discussion. |
|  | 5.3. Are the outputs of the integration of qualitative and quantitative components adequately interpreted? | Yes | Qualitative and quantitative findings are considered together to support an overall interpretation of the children’s experiences with the intervention. |
|  | 5.4. Are divergences and inconsistencies between quantitative and qualitative results adequately addressed? | Yes | No divergences or inconsistencies were identified. |
|  | 5.5. Do the different components of the study adhere to the quality criteria of each tradition of the methods involved? | Yes | Both the qualitative and quantitative components are well described and meet their quality criteria. |
|  | **Number of criteria met:** | **5/5 = Good quality** |  |
| Eklund et al., 2022c | **Screening questions (for all types):** |  |  |
|  | S1. Are there clear research questions? | Yes | The research question is clearly stated in the aim, which is to explore how the family perceived information and communication about the imminent death during the illness trajectory and after the loss. |
|  | S2. Do the collected data allow to address the research questions? | Yes | Study-specific questionnaires collected post-loss and field notes from the intervention provide relevant data to address the research question. |
|  | **Criteria of chosen category:** |  |  |
|  | 5.1. Is there an adequate rationale for using a mixed methods design to address the research question? | Yes | The reason for conducting a mixed methods study is explained. |
|  | 5.2. Are the different components of the study effectively integrated to answer the research question? | Yes | Integration of qualitative and quantitative findings occurs both in the results section and the discussion section. |
|  | 5.3. Are the outputs of the integration of qualitative and quantitative components adequately interpreted? | Yes | Qualitative and quantitative findings are interpreted together to build a comprehensive understanding of how families experienced communication about death. |
|  | 5.4. Are divergences and inconsistencies between quantitative and qualitative results adequately addressed? | Yes | No divergences or inconsistencies were identified. |
|  | 5.5. Do the different components of the study adhere to the quality criteria of each tradition of the methods involved? | Yes | Both the qualitative and quantitative components are well described and meet their quality criteria. |
|  | **Number of criteria met:** | **5/5 = Good quality** |  |
